# Supplementary figures and images for: Early detection of Cercospora beticola and powdery mildew diseases in sugar beet using uncrewed aerial vehicle-based remote sensing and machine learning
Source: PeerJ. 2025 Jun 3;13:e19530. doi: 10.7717/peerj.19530 (PMC12143290; doi:10.7717/peerj.19530)

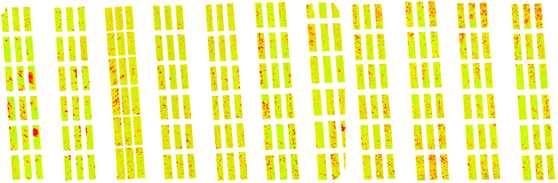

Supplement: Supplemental Information 15 [file peerj-13-19530-s015.png]

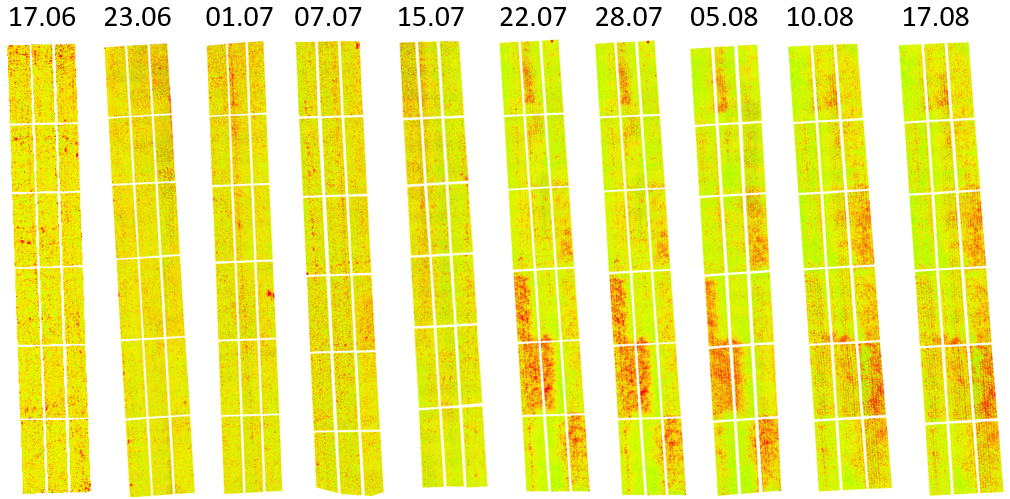

Supplement: Supplemental Information 16 [file peerj-13-19530-s016.png]

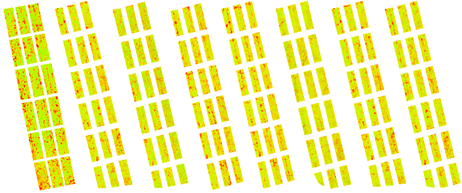

Supplement: Supplemental Information 17 [file peerj-13-19530-s017.png]

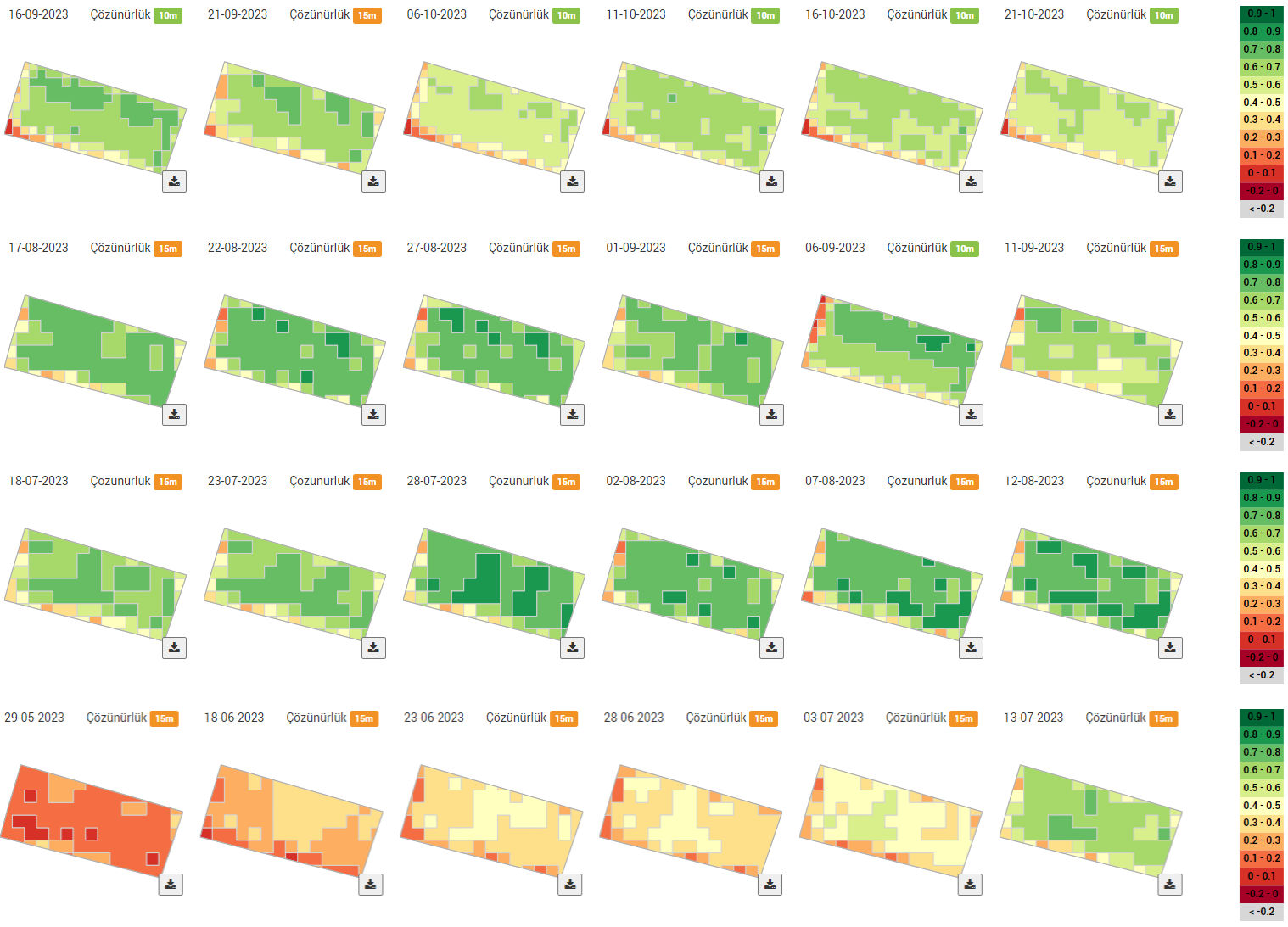

Supplement: Supplemental Information 18 [file peerj-13-19530-s018.png]

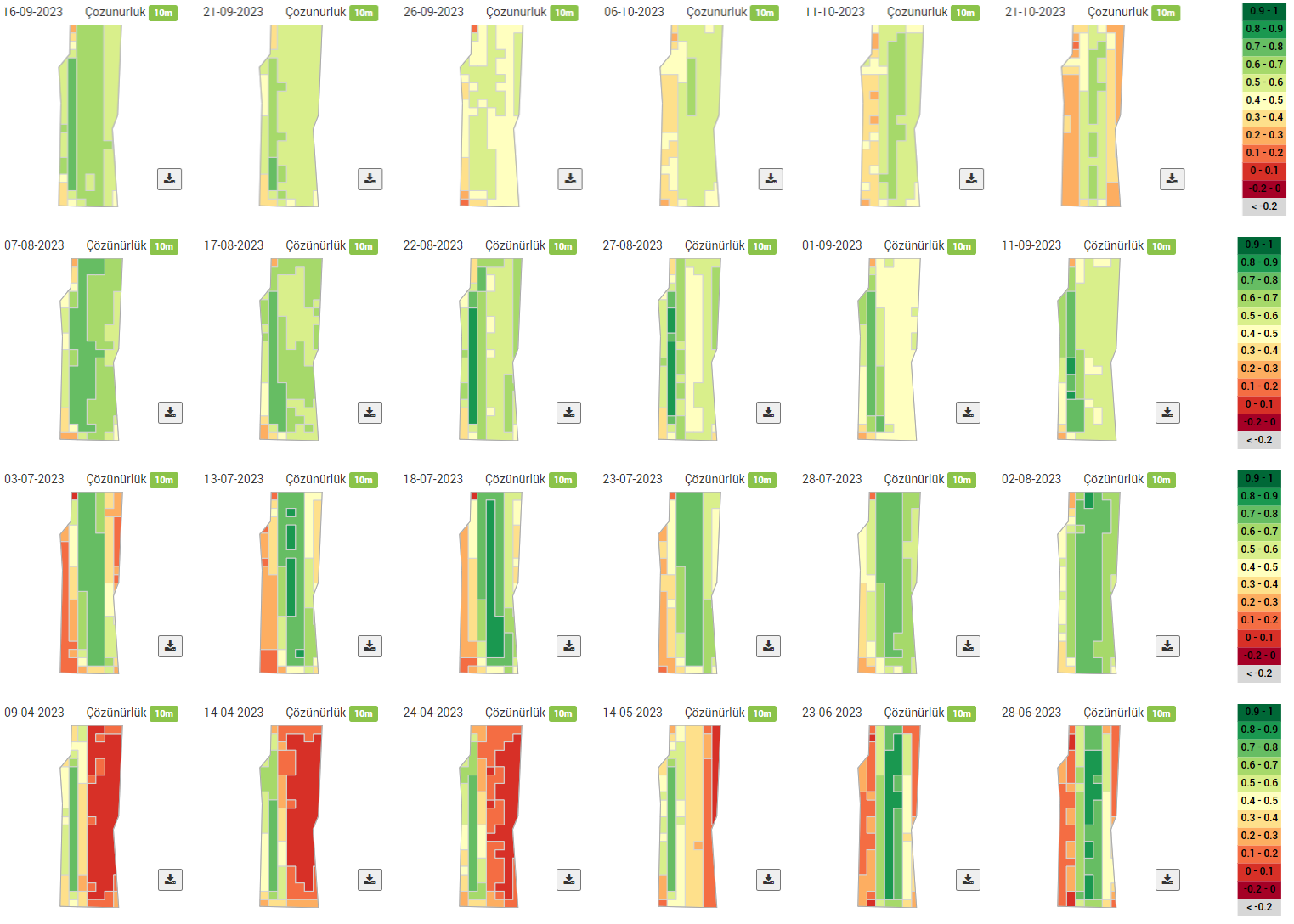

Supplement: Supplemental Information 19 [file peerj-13-19530-s019.png]

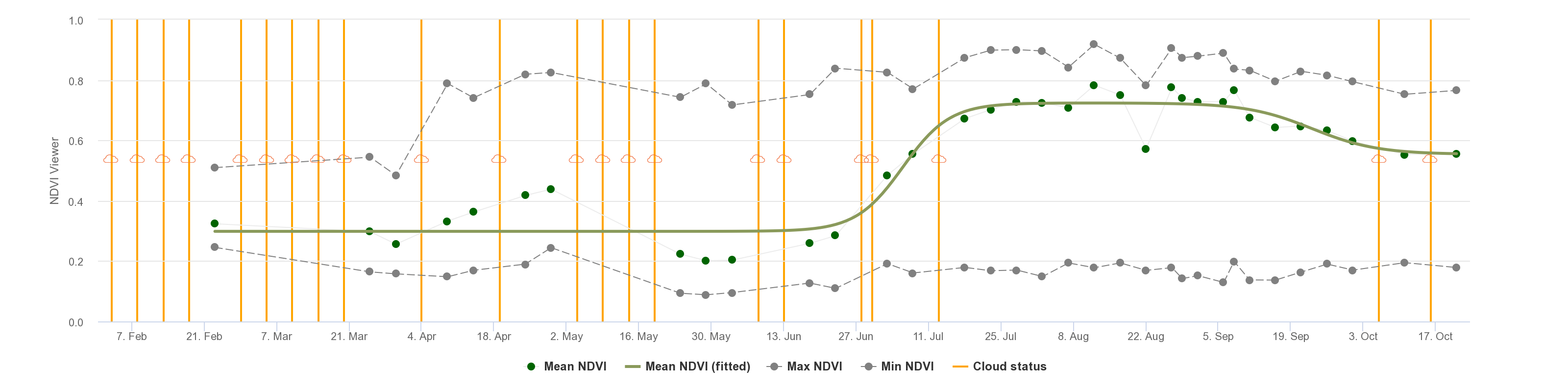

Supplement: Supplemental Information 20 [file peerj-13-19530-s020.png]

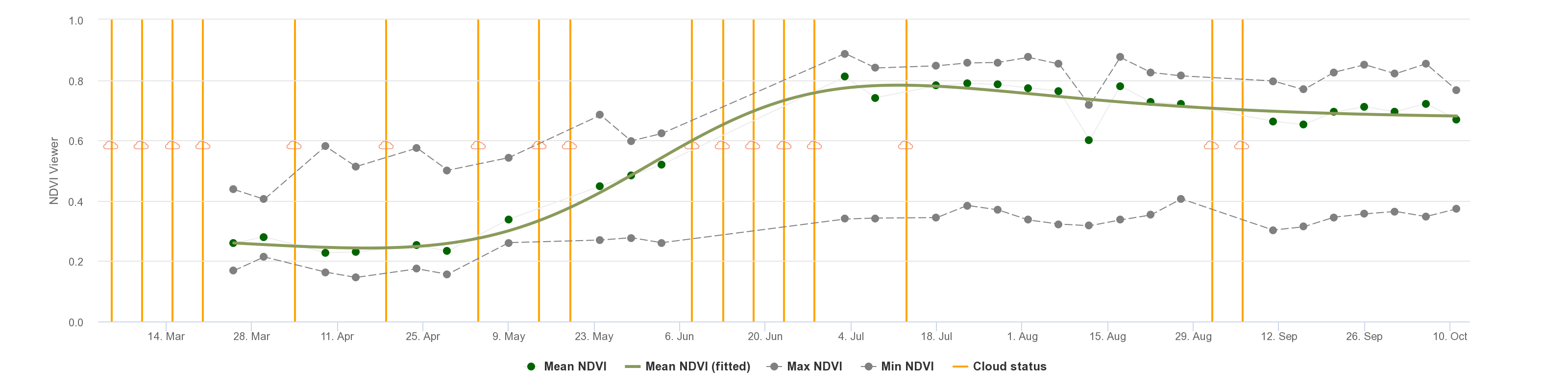

Supplement: Supplemental Information 21 [file peerj-13-19530-s021.png]

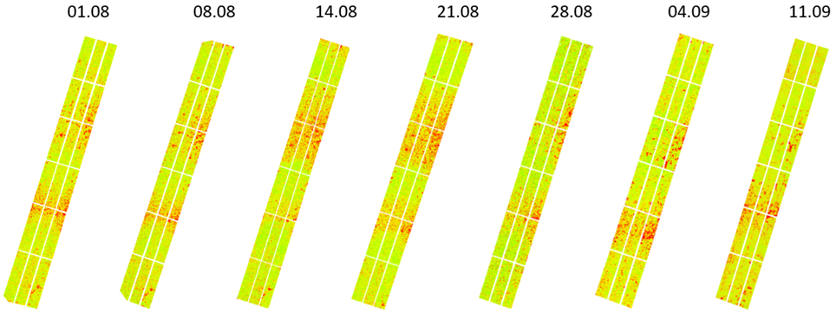

Supplement: Supplemental Information 22 [file peerj-13-19530-s022.png]
